# Single-domain antibodies as potent inhibitors of clinically relevant *β*-lactamases in multidrug-resistant bacteria

**DOI:** 10.3389/fmicb.2026.1840552

**Published:** 2026-06-10

**Authors:** Paloma Osset-Trenor, Markus Proft, Amparo Pascual-Ahuir

**Affiliations:** 1 Centro de Investigación e Innovación en Bioingeniería Ci2B, Universitat Politècnica de València, Ciudad Politécnica de la Innovación, Valencia, Spain; 2 Department of Metabolism, Inflammation and Aging, Instituto de Biomedicina de Valencia IBV-CSIC, Valencia, Spain; 3 Valencia Biomedical Research Foundation, Centro de Investigación Príncipe Felipe (CIPF) – Associated Unit to the Instituto de Biomedicina de Valencia IBV-CSIC, Valencia, Spain

**Keywords:** antibiotics, antimicrobial resistance, inhibitor, single-domain antibody, *β*-lactamase

## Abstract

Antimicrobial resistance (AMR) represents a critical threat to global health, largely driven by the dissemination of *β*-lactamases that inactivate frontline antibiotics. Among the most problematic are BlaMab-2 from *Mycobacterium abscessus*, KPC-2 and OXA-48 from *Klebsiella pneumoniae*, and VIM-2 from *Pseudomonas aeruginosa*, which together confer broad resistance to *β*-lactams and carbapenems. Current *β*-lactamase inhibitors face declining efficacy as resistance variants continue to emerge, underscoring the need for innovative strategies. Here, we explored single-domain antibodies (sd-Abs) as enzyme-directed inhibitors of *β*-lactamases. A library of sd-Abs was screened, and two candidates, B2 and B5, were characterized *in vitro* and cell-based assays. Both sd-Abs inhibited BlaMab-2 activity in *E. coli* expression systems, following a competitive inhibition mechanism, with B2 consistently displaying stronger potency (K_i_ ≈ 1.5 μM) than B5. Remarkably, B2 also demonstrated broad inhibitory activity against KPC-2, VIM-2, and OXA-48, while B5 showed an alternative inhibition profile, including uncompetitive characteristics against VIM-2 and OXA-48. Comparison with clinically deployed inhibitors revealed that the K_i_ values of B2 and B5 are of the same order of magnitude, or superior in some cases, highlighting their therapeutic promise. Our findings establish sd-Abs as a versatile platform for the inhibition of diverse *β*-lactamases, with B2 emerging as the most broadly effective candidate. By expanding the utility of existing *β*-lactams, sd-Abs could help restore antibiotic efficacy against multidrug-resistant pathogens. This study underscores the potential of antibody-based enzyme inhibitors as a new class of anti-resistance therapeutics.

## 1 Introduction

Antibiotic resistance is one of the most urgent global health challenges of our time. A landmark study published in *The Lancet* estimated that nearly 5 million deaths per year are associated with antibiotic-resistant bacterial infections, with the highest burden in low- and middle-income countries. This series also highlighted how this threat affects individuals across the entire life course, with newborns, older adults, and people with chronic conditions being particularly vulnerable (Murray et al., 2022). Beyond the health impact, antimicrobial resistance (AMR) threatens to impose a catastrophic economic toll: at current rates of inaction, treatment of resistant infections alone is projected to cost $412 billion per year by 2035, while losses in labor productivity may exceed $443 billion annually (Global Leaders Group on Antimicrobial Resistance, 2024).

*β*-lactam antibiotics are the most widely prescribed class of antibacterial agents due to their broad-spectrum activity. These compounds are characterized by a four-membered *β*-lactam ring, that covalently binds to penicillin-binding proteins (PBPs), thereby inhibiting the final steps of peptidoglycan cross-linking in the cell walls of Gram-negative and Gram-positive bacteria (Tipper and Strominger, 1965; Blumberg and Strominger, 1974; Tooke et al., 2019). Although bacteria employ multiple mechanisms to evade antibiotic action, the production of *β*-lactamases remains the most prevalent and clinically significant, especially in Gram-negative pathogens. These enzymes catalyse the hydrolysis of the *β*-lactam ring of penicillins, cephalosporins, monobactams and carbapenems, nullifying their antibacterial efficacy.

According to Ambler’s classification, which is based on sequence homology and fundamental differences in the hydrolytic mechanism, *β*-lactamases are divided into four classes: A, B, C, and D. Specifically, classes A, C, and D share the presence of a serine residue at the enzyme’s active site (serine *β*-lactamases; SBL), whereas class B consists of a heterogeneous group of zinc-dependent metalloenzymes (metallo-*β*-lactamases or MBL). These four classes are widely distributed, however, certain enzyme families have become especially prevalent among clinically significant pathogens, mainly Gram-negative bacteria responsible for opportunistic infections associated with medical care of immunocompromised patients. These key enzyme families include TEM (Temoneira), SHV (sulfhydryl variable), CTX-M (cefotaximase-Munich), and KPC (*Klebsiella pneumoniae* carbapenemase) enzymes from class A; NDM (New Delhi metallo-*β*-lactamase) and VIM (Verona integron-encoded metallo-*β*-lactamase) from class B; and CMY (cephamycinase) and ADC (*Acinetobacter*-derived cephalosporinase) from class C. Class D enzymes are called oxacillinases (OXA) and include the OXA-23 and 24/40 groups, as well as OXA-48, which are particularly concerning due to their role in carbapenem resistance in *Acinetobacter baumannii* and *Enterobacteriaceae*, respectively (Tooke et al., 2019).

**Figure 1:**
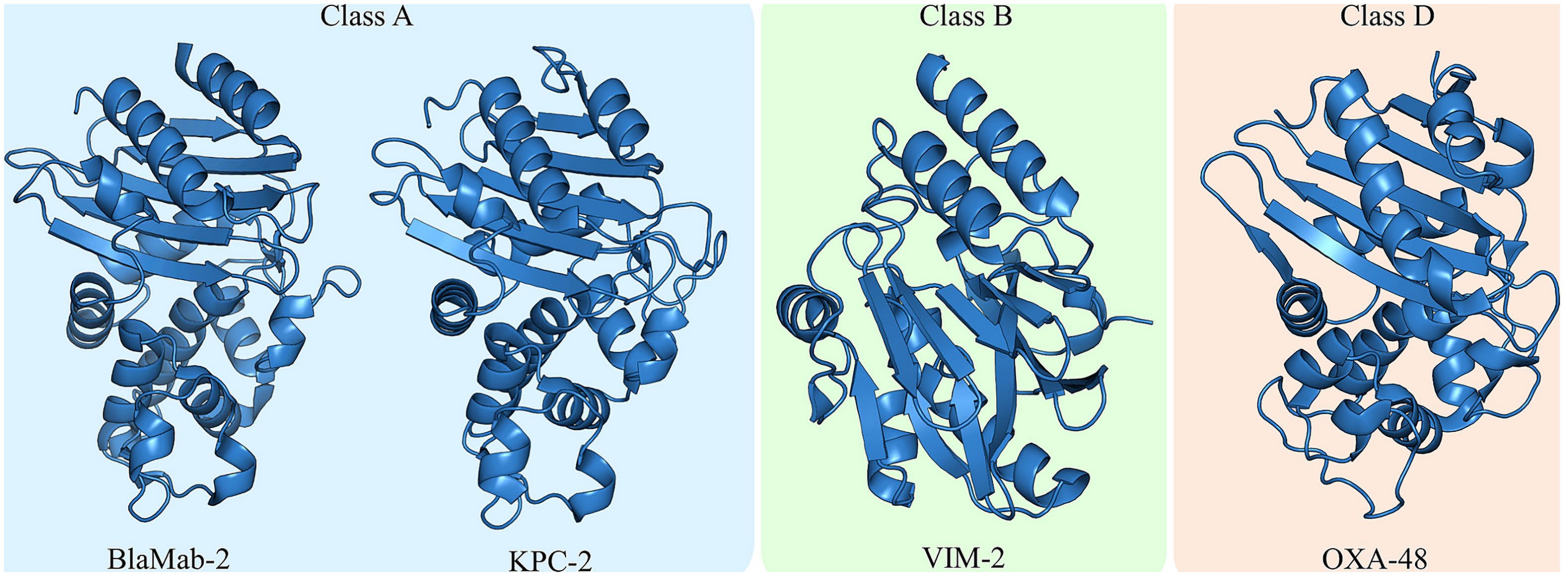
Structural representations of clinically relevant β-lactamases targeted in this study. Cartoon representations of the *β*-lactamase enzymes BlaMab-2 (*Mycobacterium abscessus*, PDB ID: 4YFM), KPC-2 (*Klebsiella pneumoniae*, PDB ID: 5UL8), OXA-48 (*Klebsiella pneumoniae*, PDB ID: 5DTK), and VIM-2 (*Pseudomonas aeruginosa*, PDB ID: 4BZ3) selected as representatives of Ambler classes A, D, and B. Structures were visualized in PyMOL using a uniform color scheme. Only one chain is shown per enzyme for clarity.

Our study focuses on representatives of the three most clinically relevant *β*-lactamase families: class A (BlaMab-2 from *Mycobacterium abscessus* and KPC-2 from *Klebsiella pneumoniae*), class D (OXA-48 from *Klebsiella pneumoniae*), and class B (VIM-2 from *Pseudomonas aeruginosa*) (Figure 1). *Mycobacterium abscessus* is a non-tuberculous mycobacterium (NTM) ranked as the second most important pathogen in NTM-associated pulmonary diseases (Cristancho-Rojas et al., 2024). Its broad resistance to antibiotics is largely attributed to its low-permeability cell envelope (Nguyen et al., 2024) and the production of the BlaMab *β*-lactamase (Dubée et al., 2014), whose deletion increases susceptibility to *β*-lactams such as amoxicillin and ceftaroline (Lefebvre et al., 2016). *Klebsiella pneumoniae* and *Pseudomonas aeruginosa* are Gram-negative pathogens listed by the World Health Organization as “critical priority” for new antibiotic development (World Health Organization, 2024). In *K. pneumoniae*, resistance is partly mediated by carbapenemases such as KPC-2 (*Klebsiella pneumoniae* carbapenemase) and the carbapenem-hydrolysing oxacillinase OXA-48 (Poirel et al., 2004). *P. aeruginosa* produces the Verona integron-encoded metallo-*β*-lactamase VIM-2, which efficiently hydrolyses carbapenems (Fortunato et al., 2023), and it is further distinguished by its capacity to form biofilms, significantly complicating treatment.

Collectively, these organisms represent important nosocomial pathogens, capable of causing opportunistic infections in immunocompromised patients, and exhibit multidrug resistance profiles (World Health Organization, 2024). Their capacity to evade multiple lines of antimicrobial therapy represents a growing challenge in intensive care units and in patients with significant comorbidities.

Given the escalating threat of AMR, it is crucial to develop novel therapeutic strategies that either directly combat resistant bacteria or enhance the efficacy of existing antibiotics. Phage therapy, which employs highly specific lytic bacteriophages to lyse pathogens without damaging the commensal microbiota (Viertel et al., 2014), has demonstrated efficacy in both preclinical models and clinical case series of refractory infections and biofilms (Gordillo Altamirano and Barr, 2019). Although its combination with antibiotics can produce synergistic effects and reduce the selection of resistant mutants (Comeau et al., 2007; Knezevic et al., 2013), phage therapy is also affected by bacterial resistance (Gordillo Altamirano and Barr, 2019). In parallel, phage-derived endolysins are hydrolases produced by phages that degrade the peptidoglycan wall of Gram-positive and Gram-negative bacteria with high specificity. In addition, they can be designed or fused with penetration peptides to act more efficiently (Schmelcher et al., 2012) and have established themselves as therapeutic candidates with potent bactericidal activities and a low resistance profile (Rahman et al., 2021). In this context, single-domain antibodies (sd-Abs) stand out for their small size (~15 kDa), high stability, and ability to be designed with high affinity and specificity toward critical enzyme targets (Muyldermans, 2013). In addition, they can be obtained through directed evolution by random mutagenesis and selection from combinatorial libraries (through phage display or yeast surface display). These properties, combined with their efficient production in bacterial systems, facilitate their design as bacterial enzyme inhibitors, including the specific inhibition of *β*-lactamases. Thus sd-Abs are positioned as particularly promising candidates for combating multidrug-resistant bacteria, including those addressed in this study (Boder and Wittrup, 1997; Muyldermans, 2013).

Several *β*-lactamase inhibitors are co-administered with *β*-lactam antibiotics to enhance their efficacy (Tooke et al., 2019). However, bacteria continuously evolve mechanisms to evade these inhibitors, leading to an increasing number of resistant strains. The aim of this study is to demonstrate the ability of two sd-Abs to neutralize *β*-lactamases from multidrug-resistant bacteria, specifically BlaMab-2, evaluate their potential as enzymatic inhibitors and determine their efficacy against other clinically relevant *β*-lactamases, including KPC-2, VIM-2 and OXA-48 from different bacterial species.

## 2 Materials and methods

### 2.1 Cloning

To obtain *E. coli* strains producing the four *β*-lactamases, synthetic DNA sequences for BlaMab-2 (PDB ID: 4YFM), KPC-2 (PDB ID: 5UL8), VIM-2 (PDB ID: 4BZ3), and OXA-48 (PDB ID: 5DTK) were obtained from Twist Bioscience. These sequences were amplified by PCR using the primers A (forward, 5’-CAATCCGCCCTCACTACAACCG) and B (reverse, 5′- TCCCTCATCGACGCCAGAGTAG). The amplified products were cloned into the pET-26b(+) vector via enzymatic digestion using the *Bam*HI restriction enzyme and subsequently transformed into *E. coli* BL21(DE3) cells (Thermo Scientific), following the manufacturer’s protocol.

A collection of 96 sd-Abs was screened, and the seven best candidates (B1-B7) were selected based on their affinity and specificity. The DNA sequences encoding the sd-Abs were amplified by PCR using the primers C (forward, 5’-CCCTC**GGATCC**GCAGGTGCAGCTGCAGGAAAGCGGC) and D (reverse, 5′- CCCC**GGATCC**GAGCTGCTCACGGTCACCTGGGTGCC) adding *Bam*HI (bold) restriction sites. The cloning into the pET-26b(+) plasmid was confirmed by colony PCR and sequencing (performed by Macrogen).

### 2.2 Expression and purification of recombinant proteins

For the production of BlaMab-2 and the sd-Abs, each recombinant strain (*E. coli* BL21(DE3) harboring the respective plasmid) was grown in LB medium supplemented with 50 μg/mL kanamycin at 37 °C overnight with agitation (200 rpm). The following day, the medium was changed to fresh LB medium and expression was induced with 1 mM *β*-D-1-thiogalactopyranoside (IPTG) at 16 °C overnight with agitation. For protein extraction, cells were centrifuged at 3400 x g for 20 min and the pellet was resuspended in sucrose solution (10 mM Tris–HCl pH 8, 1 M EDTA, 25% (w/v) Sucrose) at five times the cell pellet weight. After 10 min incubation at room temperature, cells were centrifuged at 14000 x g for 45 min. The resulting pellet was resuspended in a cold shock solution (10 mM Tris–HCl pH 8, 0.5 M MgCl_2_), incubated on ice for 10 min and centrifuged again at 14000 x g for 25 min. The supernatant containing the protein fraction, was collected and stored at 4 °C. The extraction step was repeated to maximize protein yield. Proteins were filtered through a 22 μm pore filter and purified using affinity chromatography with a HisTrap™ HP column (Cytiva). Proteins were eluted with 500 mM imidazole. BlaMab-2 was stored in 50% glycerol at −20 °C, while sd-Abs were stored at 4 °C.

### 2.3 Verification of protein production

The expression of the four *β*-lactamases was confirmed by sodium dodecyl sulphate polyacrylamide gel electrophoresis (SDS-PAGE) and Western blot analysis. For SDS-PAGE, 15% gels were used, and proteins were denatured by heating at 96 °C in loading buffer (7.5% SDS, 0.1 M dithioerythritol, 10 mM EDTA, 30% sucrose (w/v), 0.25 mg/mL bromophenol blue and 0.3 M Tris, adjusted to pH 6.8 with HCl). The PageRuler™ Prestained Protein Ladder (Thermo Scientific) was used as a molecular weight marker. Electrophoresis was performed at 120 V in running buffer (192 mM glycine and 0.1% SDS brought to pH 8.3 with Tris), followed by staining with BlueSafe protein staining reagent (NZYtech) for 15 min and destained with distilled water under gentle agitation.

For Western blot analysis, proteins were transferred onto polyvinylidene difluoride (PVDF) membranes using the Bio-Rad Western blot transfer system at 30 V overnight. Membranes were blocked with 2% skimmed milk powder in Tris-buffered saline (TBS-M) for 30 min, then incubated with anti-His-tag rabbit primary antibody (Jackson InmunoResearch Laboratories, Inc.) (1:1000 dilution in TBS-M) for 1 h. After washing, membranes were incubated with anti-rabbit-ECL secondary antibody (Cytiva) (1:5000 dilution in TBS-L) for 30 min. Protein detection was performed using the ImageQuant 800 system.

### 2.4 *In vitro* enzyme activity assays of BlaMab-2 and inhibition by B2, B5 and B7

The activity of purified BlaMab-2 was assessed using the chromogenic *β*-lactam substrate nitrocefin, a yellow chromogenic substrate that is hydrolysed by *β*-lactamases to produce a red product detectable at a wavelength of 490 nm (Spicer et al., 2010). BlaMab-2 was incubated with increasing concentrations of nitrocefin (20 μM, 40 μM, 60 μM, 80 μM, 100 μM, and 200 μM) diluted in sterile milli-Q water. The protein was used at a concentration of 9 mM and diluted 10^4^ times in phosphate buffer saline (PBS) plus 0.2% of BSA (PBSA) at pH 5.8. The hydrolysis reaction by BlaMab-2 was monitored by measuring the absorbance at 490 nm every 60s using a TECAN Infinite 200 PRO microplate reader. For inhibition assays, BlaMab-2 (same concentration as in the previous assay) was incubated with the sd-Abs (B2, B5 and B7) at a final concentration of 2 μM for 1 h at 4 °C under gentle agitation. Nitrocefin hydrolysis was then assessed as described above. Each assay was performed in triplicate.

### 2.5 Whole-cell enzyme activity assays of BlaMab-2, KPC-2, VIM-2 and OXA-48 and their inhibition by B2 and B5

The enzymatic activity of BlaMab-2, KPC-2, VIM-2, and OXA-48 was evaluated in a cellular context using recombinant *E. coli* BL21(DE3) strains. Cells were grown to an OD_600_ of 0.6–0.8 and the protein expression was induced with IPTG overnight. Cells were collected by centrifugation and the pellet was resuspended in PBSA at pH 7.4 and diluted 100-fold. Nitrocefin was added at increasing concentrations (20 μM, 40 μM, 60 μM, 80 μM, 100 μM, and 200 μM) diluted in sterile H_2_O milli-Q and the absorbance at 490 nm was measured every 45 s using a TECAN Infinite 200 PRO microplate reader. For cell-based inhibition assays, bacterial cells were incubated with B2 and B5 at a final concentration 20 μM for 1 h at room temperature before adding nitrocefin. Enzyme activity was assessed as described above.

### 2.6 Determination of kinetic parameters

The concentration of hydrolyzed nitrocefin (c) was calculated from absorbance (A), the molar extinction coefficient of hydrolyzed nitrocefin (*ε* = 15,900 M^−1^·cm^−1^, reported at 500 nm and assumed equivalent at 490 nm), and the fixed optical pathlength of the plate reader (l = 0.10 cm) using the Beer–Lambert law:


A=ε⋅c⋅l

Initial velocities ( $V_{0}$ ) were obtained from the slope of the absorbance at 490 nm versus time during the initial linear phase of the assay and converted to concentration per unit time according to:


$$V_{o}=\frac{\frac{\mathrm{\Delta A}}{\mathrm{\Delta t}}}{\varepsilon \cdot l}$$

To determine the kinetic constants $K_{m}$ and $V_{\max}$ , assays were performed at multiple substrate concentrations and $V_{0}$ values were fitted to the Michaelis–Menten equation:


$$V_{o}=\frac{V_{\max}[S]}{K_{m}+[S]}$$

using the double reciprocal Lineweaver-Burk transformation (Lineweaver and Burk, 1934), plotting $\frac{1}{V_{0}}$ (where $V_{0}$ is the initial velocity) versus $\frac{1}{\left[S\right]}$ (where [S] is the nitrocefin concentration). $K_{m}$ and $V_{\max}$ values are reported with standard error, and linear regressions were performed using GraphPad Prism 8 software.

For the estimation of the inhibition constant ( $K_{i}$ ) under the assumption of a competitive inhibition model, the following relationship was applied:


$$K_{m\;\mathrm{app}}=K_{m}(1+\frac{\left[I\right]}{K_{i}})$$

which rearranges to:


$$K_{i}=\frac{K_{m}[I]}{K_{m\;\mathrm{app}}-K_{m}}$$

where $K_{m\;\mathrm{app}}$ corresponds to the apparent $K_{m}$ value in the presence of the inhibitor, and [I] represents the inhibitor concentration, in this case, the antibody.

### 2.7 Patent statement

The single-domain antibodies (sd-Abs) used in this study, including B2 and B5, are covered by patent application P202330506, held by the Universidad Politécnica de Valencia (UPV). The materials were used for research purposes only in the present work.

## 3 Results

### 3.1 Verification of the expression of *β*-lactamases and single-domain antibodies

**Figure 2:**
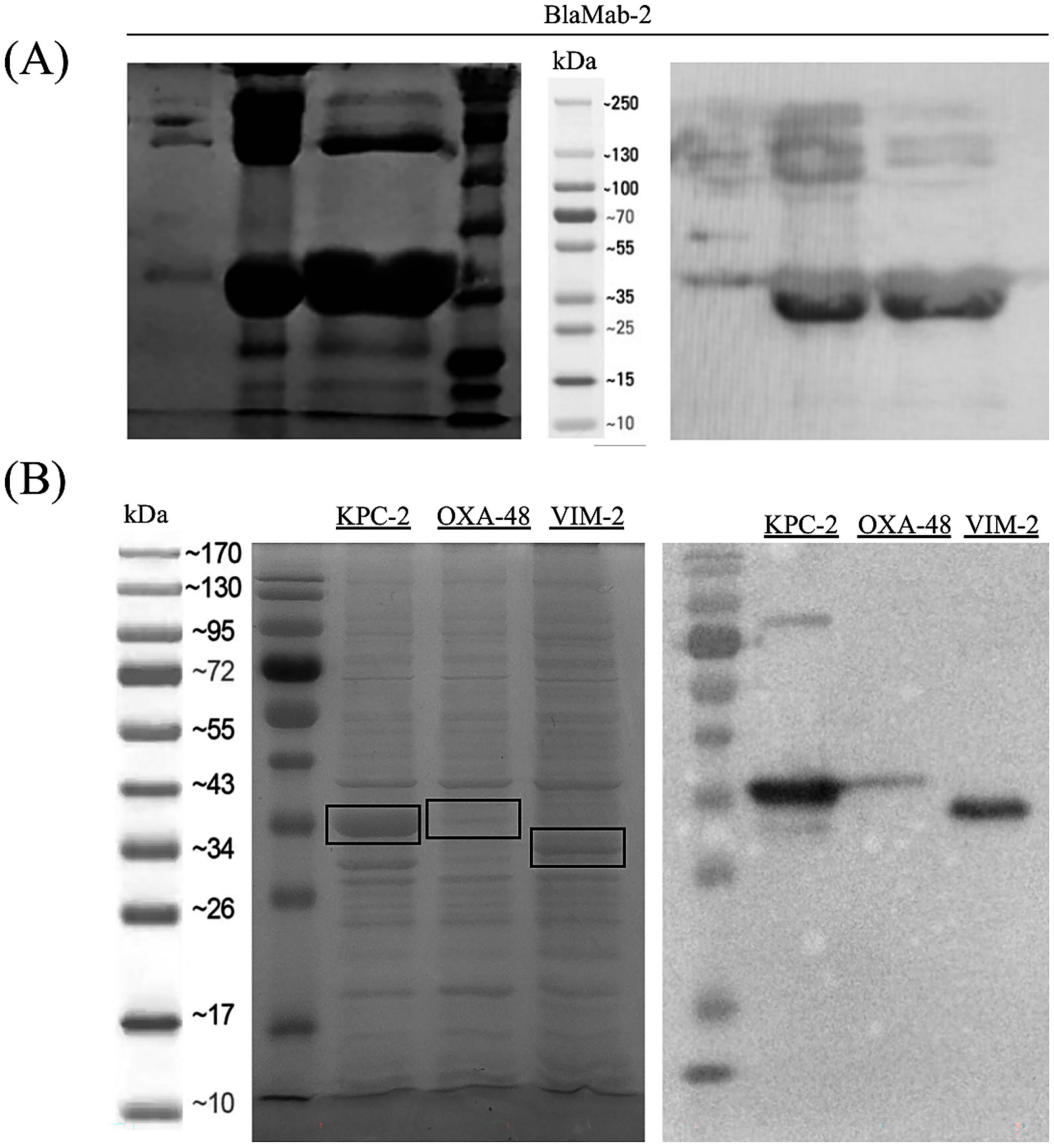
SDS-PAGE and western blot analysis of *β*-lactamases. **(A)** SDS-PAGE electrophoresis gel of three clones of BlaMab-2 after affinity purification (left) and western blot (anti-His) of the same clones (right). **(B)** SDS-PAGE electrophoresis of protein extracts from KPC-2, VIM-2, and OXA-48 expressing bacteria (left) and western blot (anti-His) confirming the presence of the three *β*-lactamases (right).

The successful heterologous expression of the four *β*-lactamases selected for this study - BlaMab-2, KPC-2, VIM-2, and OXA-48 - was first verified by SDS-PAGE and Western blot analysis. Following induction in *E. coli* BL21(DE3) and affinity purification, Coomassie-stained SDS-PAGE gels revealed prominent protein bands at molecular weights consistent with the predicted sizes of each enzyme (BlaMab-2: 33.7 kDa; KPC-2: 33.2 kDa; VIM-2: 31.4 kDa; OXA-48: 33.4 kDa), indicating efficient expression and purification (Figure 2A). Western blot analysis using an anti-His-tag antibody further confirmed the identity of these bands, demonstrating strong and specific immunoreactivity at the expected positions (Figure 2B).

In parallel, the expression of the selected sd-Abs was confirmed using the same analytical approaches (data not shown), ensuring that both enzymes and inhibitors were produced at sufficient levels and with appropriate integrity to support subsequent functional analyses.

### 3.2 Enzymatic activity of recombinant *β*-lactamases

**Figure 3:**
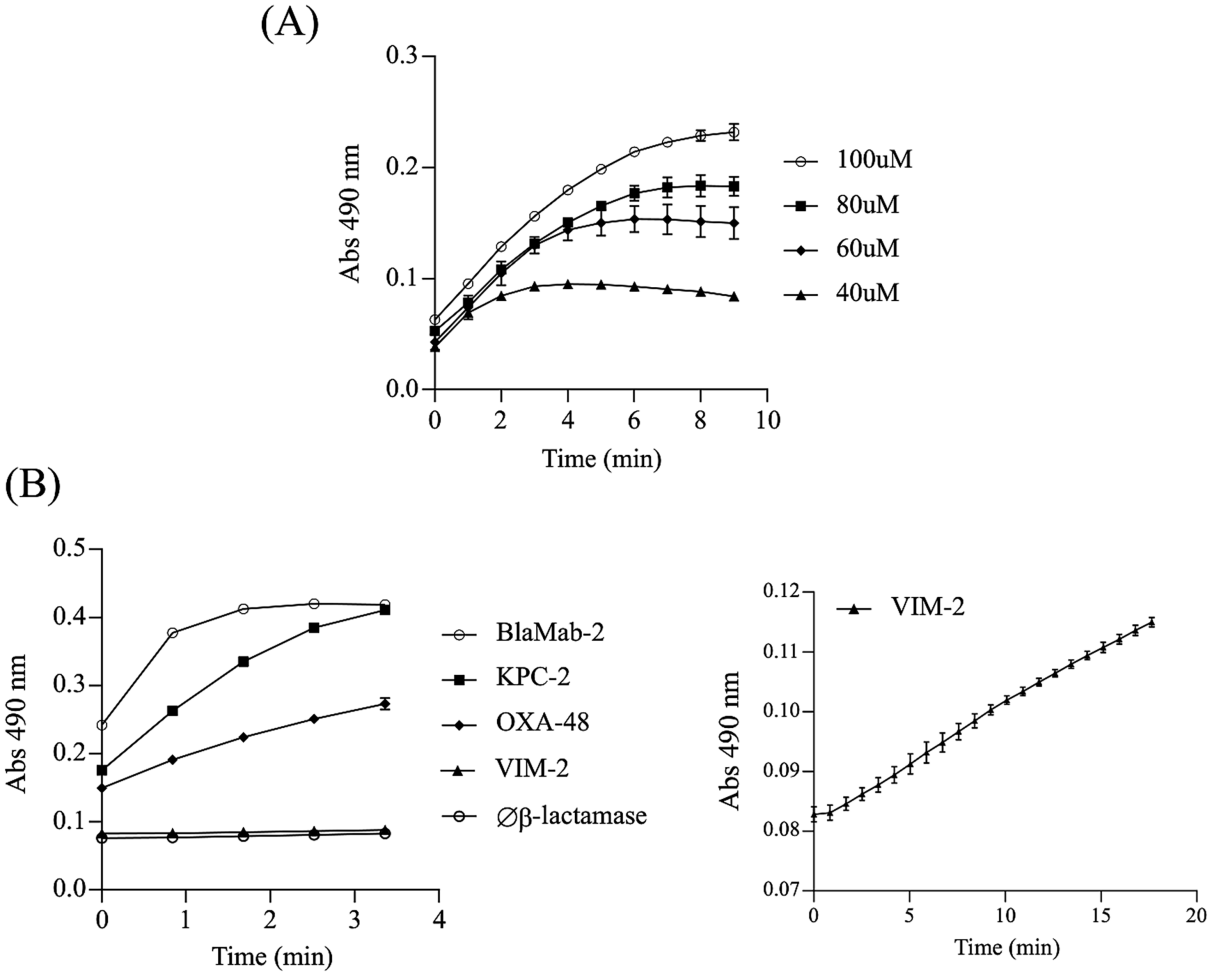
Enzymatic activity assays. **(A)** *In vitro* enzymatic activity of BlaMab-2 measured by the hydrolysis of nitrocefin at increasing concentrations at 490 nm. **(B)** Left panel: whole-cell activity of BlaMab-2, KPC-2, OXA-48, and VIM-2 in *E. coli* strains through detection of the absorbance at 490 nm produced by the hydrolysis of nitrocefin at a final concentration of 100 μM. Right panel: whole-cell activity of VIM-2 at longer incubation times.

The catalytic activity of purified BlaMab-2 was first evaluated *in vitro* using nitrocefin as a chromogenic substrate. Upon incubation with increasing concentrations of nitrocefin (20–200 μM), a clear and time-dependent increase in absorbance at 490 nm was observed, confirming efficient substrate hydrolysis (Figure 3A). Reaction velocities increased proportionally with substrate concentration, consistent with Michaelis–Menten kinetics and demonstrating that the purified enzyme retained full catalytic competence.

To assess whether *β*-lactamases were also functional in a cellular context, nitrocefin hydrolysis assays were performed using intact *E. coli* BL21(DE3) cells expressing BlaMab-2, KPC-2, VIM-2, or OXA-48. At a fixed nitrocefin concentration (100 μM), all four recombinant strains showed a robust increase in absorbance at 490 nm over time (Figure 3B). Notably, VIM-2 displayed a slower and less pronounced increase in absorbance, becoming detectable only at longer incubation times compared to the other enzymes (Figure 3B). In contrast, control cells lacking *β*-lactamase expression showed no detectable activity. These results confirm that all four enzymes are catalytically active when produced in recombinant bacterial cells and accessible to extracellular substrate, enabling whole-cell kinetic analyses.

### 3.3 Kinetic characterization of BlaMab-2 and inhibition by sd-Abs *in vitro*

Detailed kinetic analyses were performed using purified BlaMab-2 to quantify the effects of sd-Abs B2, B5, and B7 on enzyme activity. Michaelis–Menten parameters were derived from Lineweaver-Burk plots generated across a range of nitrocefin concentrations. In the absence of inhibitors, BlaMab-2 displayed a K_m_ of 45.3 ± 2.5 μM and a V_max_ of 0.22 ± 0.01 μM·min^−1^ (Table 1).

**Table 1:** Enzyme activity constants K_m_, V_max_ and K_i_ of purified *β*-lactamase BlaMab-2 for the hydrolysis of nitrocefin *in vitro*, in the absence and presence of two sd-Ab inhibitors: B2 and B5.

Ø sd-Ab		B2			B5		
K_m_ μM	V_max_ μM/min	K_mi_ μM	V_max_ μM/min	K_i_ μM	K_mi_ μM	V_max_ μM/min	K_i_ μM
45.3 ± 2.5	0.22 ± 0.01	64.4 ± 2.2	0.25 ± 0.008	2.4 ± 0.08	57.3 ± 0.9	0.23 ± 0.03	3.8 ± 0.06

Preincubation of BlaMab-2 with sd-Abs B2 or B5 resulted in a marked increase in the apparent K_m_, while V_max_ values remained largely unchanged. Specifically, B2 increased the K_m_ to 64.4 ± 2.2 μM, whereas B5 increased it to 57.3 ± 0.9 μM. This kinetic signature is characteristic of competitive inhibition, indicating that both sd-Abs interfere with substrate binding, most likely by interacting directly with or near the active site of the enzyme. Consistent with this interpretation, calculated K_i_ values revealed that B2 (K_i_ = 2.4 ± 0.08 μM) is a more potent inhibitor than B5 (K_i_ = 3.8 ± 0.06 μM).

In contrast, sd-Ab B7 produced anomalous kinetic behavior, yielding a negative K_i_ value (−3.7 ± 0.6; Supplementary material). Such a value is not physically meaningful within classical inhibition models and suggests that B7 does not function as a *bona fide* inhibitor under the experimental conditions tested. This behavior may reflect weak or non-specific interactions, or a possible allosteric effect that enhances enzyme activity rather than inhibiting it.

### 3.4 Cell-based inhibition of *β*-lactamases by sd-Abs B2 and B5

To evaluate the inhibitory potential of sd-Abs in a physiologically relevant context, whole-cell nitrocefin hydrolysis assays were performed using *E. coli* strains expressing each *β*-lactamase. In the absence of inhibitors, apparent K_m_ values varied considerably among enzymes, reflecting intrinsic differences in catalytic efficiency and substrate affinity (Table 2 and Table 3).

**Table 2:** Enzyme activity constants K_m_, V_max_ and K_i_ of the *β*-lactamases BlaMab-2, KPC-2, VIM-2 and OXA-48 produced in recombinant *E. coli* BL21(DE3) cells, for the hydrolysis of nitrocefin, in the absence and presence of the sd-Abs inhibitors B2 and B5.

***β***-lactamase	Ø sd-Ab	B2		B5	
	K_m_ μM	K_mi_ μM	K_i_ μM	K_mi_ μM	K_i_ μM
BlaMab-2	48.029 ± 1.28	110.11 ± 2.068	1.52 ± 0.028	159.53 ± 2.015	5.90 ± 0.074
KPC-2	1.36 ± 0.11	120.91 ± 2.73	0.060 ± 0.0014	2.19 ± 3.62	0.12 ± 0.0068
VIM-2	4.35 ± 0.035	39.61 ± 0.78	2.90 ± 0.057	0.97 ± 0.026	68.92 ± 1.85
OXA-48	4.48 ± 0.062	6.62 ± 0.16	10.51 ± 0.25	2.26 ± 0.058	30.77 ± 0.79

**Table 3:** Whole-cell K_m_ values of recombinant *β*-lactamases and their inhibition by B2 and B5, compared with published in vitro data for nitrocefin hydrolysis.

***β***-lactamase	K_m_ μM (literature)	K_m_ μM	K_m_ with B2 μM	K_m_ with B5 μM
BlaMab-2	24 ± 7.0 (Soroka et al., 2014)	48.029 ± 1.28	110.11 ± 2.068	159.53 ± 2.015
KPC-2	45 ± 2.1 (Sun et al., 2024)	1.36 ± 0.11	120.91 ± 2.73	2.19 ± 3.62
VIM-2	18 (Docquier et al., 2003)	4.35 ± 0.035	39.61 ± 0.78	0.97 ± 0.026
OXA-48	23.9 ± 3.5 (Aertker et al., 2020)	4.48 ± 0.062	6.62 ± 0.16	2.26 ± 0.058

Preincubation of bacterial cells with sd-Ab B2 resulted in a substantial increase in K_m_ for all four *β*-lactamases tested. For BlaMab-2, the K_m_ increased from 48.0 ± 1.3 μM to 110.1 ± 2.1 μM, corresponding to a K_i_ of 1.52 ± 0.03 μM. Even more pronounced effects were observed for KPC-2, where B2 increased the K_m_ nearly two orders of magnitude (from 1.36 ± 0.11 μM to 120.9 ± 2.7 μM), yielding a K_i_ of 0.060 ± 0.001 μM. These data indicate strong competitive inhibition by B2 across enzymes from Ambler classes A, B, and D.

The inhibitory profile of sd-ab B5 was more heterogeneous. While B5 also increased the K_m_ of BlaMab-2 and KPC-2, consistent with competitive inhibition, its effect on VIM-2 and OXA-48 differed markedly. In these cases, B5 caused a decrease in K_m_ (to 0.97 ± 0.03 μM for VIM-2 and 2.26 ± 0.06 μM for OXA-48), accompanied by relatively high K_i_ values. Although these observations may reflect alternative interaction modes between B5 and distinct *β*-lactamases, including possible substrate-dependent conformational effects, structural and biophysical studies will be required to clarify the molecular basis of these differences.

### 3.5 Comparative analysis with clinically used *β*-lactamase inhibitors

To contextualize the inhibitory potency of sd-Abs B2 and B5, their K_i_ values were compared with those reported for established *β*-lactamase inhibitors (Table 4). For BlaMab-2, B2 displayed a K_i_ comparable to that of clavulanic acid and superior to relebactam, although less potent than newer diazabicyclooctane inhibitors such as avibactam. Notably, against KPC-2, B2 exhibited K_i_ values in the same order of magnitude as vaborbactam and markedly lower than those reported for classical inhibitors such as clavulanic acid, sulbactam, and tazobactam.

**Table 4:** Comparison of K_i_ values of sd-Ab B2 and B5 for the inhibition of BlaMab-2, KPC-2, VIM-2 and OXA-48 in cell-based assays with those reported in the literature.

***β***-lactamase	K_i_ B2 μM	K_i_ B5 μM	K_i_ μM (literature)		Ref.
BlaMab-2	1.52 ± 0.028	5.90 ± 0.074	Clavulanic acid	2.3 ± 0.7	Ramírez et al. (2017)
			Durlobactam	(4.0 ± 0.8) × 10^−3^	Dousa et al. (2022)
			Avibactam	0.30 ± 0.03	Dousa et al. (2020)
			Relebactam	136 ± 14	Dousa et al. (2020)
KPC-2	0.060 ± 0.0014	0.12 ± 0.0068	Clavulanic acid	29.96	Khan et al. (2014)
			Sulbactam	23.21	Khan et al. (2014)
			Tazobactam	21.61	Khan et al. (2014)
			Taniborbactam	0.004 ± 0.001	Hamrick et al. (2020)
			Avibactam	0.0056 ± 0.0007	Hamrick et al. (2020)
			Vaborbactam	0.022 ± 0.002	Hamrick et al. (2020)
VIM-2	2.90 ± 0.057	68.92 ± 1.85	Mitoxantrone	1,5 ± 0,2	Minond et al. (2009)
			Taniborbactam	0.019 ± 0.001	Hamrick et al. (2020)
OXA-48	10.51 ± 0.25	30.77 ± 0.79	Taniborbactam	0.35 ± 0.007	Hamrick et al. (2020)
			Avibactam	0.26 ± 0.005	Hamrick et al. (2020)
			Vaborbactam	0.35 ± 0.007	Hamrick et al. (2020)

For VIM-2 and OXA-48, B2 was less potent than leading broad-spectrum inhibitors such as taniborbactam, yet still demonstrated clear inhibitory activity at low micromolar concentrations. Collectively, these results indicate that sd-Ab B2 functions as a broad-spectrum *β*-lactamase inhibitor with competitive activity across multiple enzyme classes, whereas B5 displays a more enzyme-specific and mechanistically diverse inhibition profile.

Taken together, the expanded kinetic and functional analyses demonstrate that sd-Abs, particularly B2, can effectively inhibit clinically relevant *β*-lactamases both *in vitro* and *in vivo*, supporting their potential as a novel class of biologic *β*-lactamase inhibitors.

## 4 Discussion

In this study, we explored the potential of single-domain antibodies (sd-Abs) as inhibitors of clinically relevant *β*-lactamases from multidrug-resistant pathogens, with a particular focus on BlaMab-2 from *Mycobacterium abscessus* and representative enzymes from Ambler classes A (KPC-2), B (VIM-2), and D (OXA-48). By combining *in vitro* and whole-cell kinetic analyses, our work provides a comprehensive functional assessment of sd-Ab-mediated *β*-lactamase inhibition and positions these biologics within the broader landscape of anti-resistance strategies.

### 4.1 Functional expression and activity of recombinant *β*-lactamases

The successful heterologous expression of all four *β*-lactamases in *Escherichia coli* BL21(DE3) was confirmed by SDS-PAGE and Western blot analyses, yielding proteins of the expected molecular weights and sufficient purity for kinetic characterization. Nitrocefin hydrolysis assays demonstrated that purified BlaMab-2 retained full catalytic activity, following Michaelis–Menten kinetics consistent with previous reports. Although the K_m_ value obtained for BlaMab-2 (45.3 ± 2.5) was higher than some published values reported by Ramírez et al. in 2017 (29 ± 4) and Soroka et al. in 2014 (24 ± 7), such differences are likely attributable to variations in expression systems, purification procedures, or assay conditions, all of which can influence enzyme conformation and apparent substrate affinity.

Importantly, whole-cell assays confirmed that BlaMab-2, KPC-2, VIM-2, and OXA-48 are catalytically active when expressed in recombinant *E. coli* cells, validating this system as a physiologically relevant platform to evaluate inhibitor efficacy. For BlaMab-2, the close agreement between *in vitro* and *in vivo* K_m_ values suggests that the cellular environment does not markedly alter substrate recognition. For the other enzymes, direct comparison with literature values is limited by the scarcity of cell-based or *in vivo* kinetic data; nevertheless, the observed activities are consistent with efficient enzyme folding and accessibility to substrate in the bacterial periplasm.

### 4.2 Inhibitory profiles of sd-Abs B2 and B5

The study of new *β*-lactamase inhibitors is a promising and commonly adopted strategy to combat *β*-lactam antibiotic resistance (Zhou et al., 2022). Both sd-Abs characterized in this study, B2 and B5, effectively inhibited BlaMab-2, as evidenced by increased apparent K_m_ values with minimal changes in V_max_, indicative of competitive inhibition. This behavior suggests that both antibodies interfere with substrate binding, likely through interactions at or near the active site. Across all assays, B2 consistently exhibited lower K_i_ values than B5, pointing to a higher affinity and a more robust inhibitory interaction.

The analysis of sd-Ab B7, which yielded non-physiological negative Ki values, underscores the importance of rigorous kinetic validation. Rather than acting as a true inhibitor, B7 may engage in weak, nonspecific, or potentially activating interactions, possibly stabilizing the enzyme or enhancing substrate turnover. Such behavior highlights the mechanistic diversity that can arise from antibody-enzyme interactions and emphasizes the need for structural studies to distinguish inhibitory from modulatory binding modes.

A key finding of this work is the broad-spectrum activity of sd-Ab B2. In whole-cell assays, B2 increased the apparent K_m_ of all four *β*-lactamases tested, supporting a competitive inhibition mechanism across Ambler classes A, B, and D. This is particularly notable given the substantial structural and mechanistic differences between serine *β*-lactamases and metallo-*β*-lactamases. The ability of a single sd-Ab to inhibit such diverse enzymes suggests that B2 may recognize conserved structural or electrostatic features shared among *β*-lactamases, rather than class-specific catalytic residues.

In contrast, B5 displayed a more heterogeneous inhibition profile. While it behaved as a competitive inhibitor against BlaMab-2 and KPC-2, its effects on VIM-2 and OXA-48 were consistent with uncompetitive inhibition, characterized by decreased K_m_ values. This pattern suggests preferential binding to the enzyme-substrate complex, stabilizing a non-productive state. Although less common, uncompetitive inhibition may offer therapeutic advantages under conditions of high substrate concentration, where competitive inhibitors become less effective.

### 4.3 Implications for *β*-lactamase inhibition strategies

The inhibitory activity of the sd-Abs B2 and B5 was assessed by comparing their K_i_ values with those reported for established *β*-lactamase inhibitors. Conventional inhibitors such as clavulanic acid, tazobactam, sulbactam, and avibactam are increasingly compromised by resistant *β*-lactamases, underscoring the need for new strategies. In this study, both sd-Abs demonstrated highly favorable K_i_ values relative to those previously described. In the case of BlaMab-2, inhibition by sd-Ab B2 yielded a K_i_ of the same order of magnitude as clavulanic acid (Ramírez et al., 2017), while sd-Ab B5 achieved a notably lower value than relebactam (Dousa et al., 2020).

For KPC-2, sd-Ab B2 displayed a K_i_ value comparable to that reported for vaborbactam (Hamrick et al., 2020). Notably, the K_i_ values obtained for B2 and B5, were three and two orders of magnitude lower, respectively, than those previously reported for the classical *β*-lactamase inhibitors clavulanic acid, sulbactam and tazobactam (Khan et al., 2014). In the case of VIM-2, the Ki determined for B2 was slightly higher than that reported for mitoxantrone (Minond et al., 2009), yet remained within a range that supports its potential as a VIM-2 inhibitor. For OXA-48, B2 showed K_i_ values two orders of magnitude higher than those reported for taniborbactam, avibactam and vaborbactam (Hamrick et al., 2020). As with VIM-2, these results suggest that B2 may still serve as a valuable inhibitor, particularly under conditions where resistance compromises the efficacy of existing compounds. Overall, both sd-Abs demonstrated substantial inhibitory activity; however, B2 consistently outperformed B5, emerging as the most promising sd-Ab inhibitor due to its strong and consistent competitive inhibition profile across multiple *β*-lactamases and its lower K_i_ values for all four enzymes tested. Although less potent, B5 retains interest as an inhibitor with a distinct profile and warrants further investigation.

### 4.4 Limitations and future perspectives

Despite the promising inhibitory activity observed in this study, several challenges must be addressed before sd-Abs can be developed as therapeutic agents. First, further structural and biophysical studies, such as X-ray crystallography or cryo-EM, are necessary to elucidate the precise binding interactions between sd-Abs and *β*-lactamases. This information would facilitate the rational design of improved inhibitors with enhanced affinity and specificity. Second, *in vivo* studies are necessary to assess the pharmacokinetics, stability, and therapeutic efficacy of sd-Abs in infection models. Finally, large-scale production strategies must be optimized to enable cost-effective manufacturing for clinical use. Overall, the differential behavior of B2, B5, and B7 underscores the complexity of *β*-lactamase inhibition by sd-Abs and highlights the necessity of further characterization to fully exploit their therapeutic potential.

An additional aspect that warrants further investigation is the stability and bioavailability of sd-Abs under physiologically relevant conditions. Although sd-Abs are generally characterized by high thermal and proteolytic stability, their persistence and activity in complex environments such as the bacterial periplasm or infected tissues remain to be determined. Likewise, efficient delivery of sd-Abs to the site of infection represents an important challenge for future therapeutic development, particularly in systemic or biofilm-associated infections.

### 4.5 Concluding remarks

Overall, this study demonstrates that single-domain antibodies can function as effective inhibitors of diverse, clinically relevant *β*-lactamases. Among the candidates tested, sd-Ab B2 emerges as the most broadly active and mechanistically consistent inhibitor, while B5 highlights the potential for alternative modes of enzyme inhibition. These findings support the further exploration of sd-Abs as a novel class of *β*-lactamase inhibitors and reinforce their promise as tools to extend the lifespan of existing antibiotics in the fight against antimicrobial resistance.

## Data Availability

The raw data supporting the conclusions of this article will be made available by the authors, without undue reservation.
