# Supplementary material for: Single-domain antibodies as potent inhibitors of clinically relevant β-lactamases in multidrug-resistant bacteria
Source: Front Microbiol. 2026 Jun 10;17:1840552. doi: 10.3389/fmicb.2026.1840552 (PMC13291037; doi:10.3389/fmicb.2026.1840552)
Supplement: Supplementary file 1 [file Table_1.DOCX]

**Supplementary Material**

Table 5. Enzyme activity constants K_m_, V_max_ and K_i_ of purified β-lactamase BlaMab-2 for the hydrolysis of nitrocefin in vitro, in the absence and presence of the sd-Ab B7.

| **Ø sd-Ab** | | **B2** | | |
| --- | --- | --- | --- | --- |
| Km µM | Vmax µM/min | Kmi µM | Vmax µM/min | Ki µM |
| 45,3 ± 2,5 | 0,22 ± 0,01 | 57,3 ± 0,9 | 0,18 ± 0,03 | -3,7 ± 0,6 |
